# A Comparative pH‐Dissolution Profile Analysis of Selected Commercial Levothyroxine Formulations in Lebanon Using Anion‐Exchange HPLC Method: Implication on Interchangeability

**DOI:** 10.1155/adpp/8833028

**Published:** 2025-09-03

**Authors:** Malak AlBathish, Azza Gazy, Marwa Al Jamal

**Affiliations:** ^1^ Department of Pharmaceutical Technology, Faculty of Pharmacy, Beirut Arab University, Beirut, Lebanon, bau.edu.lb

**Keywords:** anion-exchange HPLC, comparative analysis, dissolution, levothyroxine sodium, narrow therapeutic index

## Abstract

Hypothyroidism is a common endocrine disorder that requires medical intervention by the administration of hormone replacement therapy: levothyroxine—a drug recognized as a NTI drug. Generic levothyroxine formulations can be considered as an economic alternative; however, bioequivalence problem has been encountered between various available levothyroxine formulations; thus, generic substitution is considered controversial. Dissolution testing is often used to assess the bioequivalence. The dissolution of levothyroxine from four pharmaceutical formulations: Euthyrox (old and new formulations), Eltroxin, and generic levothyroxine Sandoz was studied using a sensitive anion‐exchange HPLC method. Dissolution profiles were compared using model‐dependent and model‐independent approaches. Results showed that there is significant difference between the formulations confirmed by the similarity (*f*_1_) and dissimilarity (*f*_2_) factors. All formulations showed variable and pH‐dependent dissolution behaviors where at pH 1.2, the highest dissolution (almost 100%) is achieved. The drug‐release kinetics model for each formulation varied depending on the dissolution media; where no unique kinetic model can be used to describe the release of levothyroxine from the tablets. The revealed variations in the in vitro dissolution profiles of the four formulations could be due to excipient variability from one brand to another affecting oral absorption and bioavailability and may be the reason behind bioequivalence problems between various formulations.

## 1. Introduction

Hypothyroidism is a common endocrine disorder caused by a depleted level of the thyroid hormones T3 and T4—known as endogenous levothyroxine. The main pharmacological treatment is via a hormonal replacement therapy by the intake of oral levothyroxine which is classified as a narrow therapeutic index (NTI) drug [1, 2]. The dosing regimen of levothyroxine as a NTI drug is critical because doses outside of the therapeutic window can pose severe side effects either due to hypo‐ or hyperthyroidism [3]. Accordingly, to maintain a normal thyroid hormonal plasma concentration, a strict and consistent dosing regimen consisting of daily intake of levothyroxine, for a long duration if not life‐long, is required [1, 2].

Ever since its introduction into the market, levothyroxine pharmaceutical formulations have faced several problems and drug recalls. These problems can be divided into struggles in achieving clinical efficacy and formulation incompatibility and variability. In regards to achieving clinical efficacy of levothyroxine, the main faced problem is the patient compliance, where daily levothyroxine dose should be strictly taken on empty stomach and not skipped as it may cause bothersome hypothyroidism symptoms [4–7]. Formulation wise, some of the marketed tablets, regardless whether the brand or generics, did not meet the requirements of content uniformity, potency specification, and chemical stability in presence of their excipients [8–15]. Thus, the national regulatory bodies issued a stricter potency specification where the content per tablet for levothyroxine should be between 95% and 105% of its labeled content [3, 16, 17]. Additionally, Research and Development departments in the manufacturing companies carefully investigated the chemical properties of excipients to be used by selecting the ones that are inert and allow the best drug release into the blood stream.

Another formulation problem is in the inadequate bioequivalence between different levothyroxine formulations that was detected when patients switched between levothyroxine formulations whether brands or generics as a cost‐cutting approach by insurance companies and in cases of drug shortages [18–21].

Therefore, an essential step to target this formulation issue is by the correct choice of the excipients of the pharmaceutical formulation. Moreover, equivalency assessment in terms of identity, purity, dose strength, quality, and bioavailability assessment via biowaiver studies is required.

Biowaiver studies often use in vitro dissolution testing as a prediction of in vivo bioavailability. During dissolution testing, the amount of drug released from the pharmaceutical formulation under conditions mimicking the gastrointestinal tract is measured as a function of time to aid in quality control and formulation development and to check for bioavailability equivalence. If a drug formulation exhibits similar dissolution profiles to the reference brand product, this suggests that they may also have similar in vivo performance in terms of absorption and bioavailability; thus, interchangeability and switching can be approved [22–24]. Regulatory agencies such as FDA, WHO, EMA, and MHRA have approved such comparison for drugs belonging to Classes I and III in the Biopharmaceutics Classification System (BCS) [24–27].

Levothyroxine was often classified as a BCS Class I drug, based on its high aqueous solubility and high permeability [28]. However, other studies contradicted this classification and considered levothyroxine as belonging to Class III. This was based on an intramolecular interaction resulting in low permeability values [29, 30]. Recently, MHRA labeled levothyroxine as belonging to neither Class I nor III. This was attributed to atypical dissolution where levothyroxine self‐associates to form aggregates, resulting in low intrinsic solubility and intrinsic dissolution rate [17].

Thus, levothyroxine does not belong to any of the 4 BCS classes. That is why, in vivo bioequivalence studies are recommended; however, they are not feasible as the exogenous levothyroxine (formulated drug) cannot be distinguished from the endogenous natural hormone.

Accordingly, in our research work, biowaiver studies are performed to test for the similarities and differences in the dissolution profiles of the uncategorized drug and to assess the formulation variability influence on the dissolution profile of different marketed levothyroxine formulations. This would allow a hypothetical assessment of the bioavailability in order to have the right decision of formulation substitution. Accurate determination of levothyroxine in different dissolution media and in the presence of excipients requires the application of a highly selective and sensitive analytical method. HPLC seems to be an appealing option as it is easily available and is able to separate, identify, and quantify levothyroxine in the dissolution media [31]. Therefore, a validated HPLC method will be employed for accurate quantitation of levothyroxine during dissolution testing of the different levothyroxine formulations.

## 2. Material and Methods

### 2.1. Chemicals and Reagents

Levothyroxine sodium CRS of 89.2% purity (European Pharmacopeia Reference Standard) was purchased and used as a reference standard.

The pharmaceutical formulations used (Euthyrox100 (old formulation), Euthyrox100 (new formulation), and Eltroxin) were purchased from a local authorized pharmacy in Lebanon while a generic levothyroxine sodium 100 μg (Sandoz Inc.) was obtained from Arbor Lane Pharmacy (MI, USA). Detailed information about the used tablets in the dissolution study are presented in Table 1; these data were available on the packages and leaflets of the formulations. Dissolution testing was performed within the expiry date of the products.

**Table 1:** Detailed description of levothyroxine sodium tablets.

Formulation	Manufacturer	Excipients	Batch no.	Expiry date
Euthyrox100® (old formulation)	Merck	Starch, croscarmellose sodium, gelatin, lactose monohydrate, magnesium stearate	G00VNR	08/2023
Euthyrox100® (new formulation)	Merck	Starch, citric acid, anhydrous croscarmellose sodium, gelatin, magnesium stearate, mannitol	G013CB	04/2024
Eltroxin®	Aspen	Microcrystalline cellulose, pregelatinized starch, talc, silica, magnesium stearate	B61019B	02/2021
Levothyroxine Sandoz	Sandoz	Starch, silicon dioxide, microcrystalline cellulose, magnesium stearate, D&C yellow No. 10, D&C red No. 27, D&C red No. 30	NA	01/2021

The organic HPLC grade solvents (methanol and acetonitrile) used were from Honeywell Riedel‐de Haën.

Reagents used were of analytical grade, including sodium hydroxide pellets (Honeywell Fluka), anhydrous sodium acetate (CH_3_COONa) (Panreac), potassium phosphate monobasic (Riedel‐de Haën), potassium chloride (Sigma‐Aldrich), acetic acid (99.8%–100.5%) (Sigma‐Aldrich), phosphoric acid (≥ 85%) (Sigma‐Aldrich), and hydrochloric acid (37%) (Merck).

Purified deionized water (resistivity ≈ 18.2 MΩ cm^−1^, filtered through a 0.2‐μm capsule filter) was prepared in‐house using the Milli Q water purification system (Siemens Labostar TWF UV 7).

### 2.2. Instrumentation

HPLC chromatographic separation was achieved on an ion exchange HiQ Sil NH_2_ column (4.00 mm × 300 mm). The HPLC system (Jasco, Japan) consisted of quaternary pump, PU‐20 89/I plus, vacuum degasser, a quaternary gradient pump, diode array and multiple wavelength detector MD 2018 plus. The liquid chromatographic system is equipped with manual injection, which uses a Rheodyne port sample injection valve and fitted with 20‐μL sample loop. All are Jasco PU‐2089 series.

Dissolution studies were performed in a Copley dissolution tester DIS 8000 (Nottingham, United Kingdom), a USP Apparatus II‐paddle system.

Ohaus Explorer analytical micro balance was used for weighing out the used powders and tablets.

An Ohaus Starter 3100 pH meter calibrated with standard buffers at room temperature was used for pH measurements.

### 2.3. Preparation of Stock Solutions and Dissolution Media

#### 2.3.1. Preparation of Levothyroxine Standard Solution (100 μg/mL)

Levothyroxine standard solution was prepared at a concentration of 100 μg/mL using 50%, v/v methanolic NaOH as solvent.

#### 2.3.2. Preparation of Dissolution Media [32]

##### 2.3.2.1. pH = 1.2

This dissolution medium was prepared by dissolving 14.914 g of potassium chloride with around 425 mL of 0.2‐M hydrochloric acid to approximately 1 L of degassed water. The pH of the solution was adjusted to 1.2 with 0.2‐M sodium hydroxide or 0.2‐M hydrochloric acid as required.

##### 2.3.2.2. pH = 4.5

This dissolution medium was prepared by dissolving 2.99 g of anhydrous sodium acetate with around 14 mL of 2‐M acetic acid to approximately 1 L of degassed water. The pH of the solution was adjusted to 4.5 with 0.2‐M sodium hydroxide or 2‐M acetic acid as required.

##### 2.3.2.3. pH = 6.8

This dissolution medium was prepared by dissolving 6.805 g of monobasic potassium phosphate and 0.896 g of sodium hydroxide to approximately 1 L of degassed water. The pH of the solution was adjusted to 6.8 with 0.2‐M sodium hydroxide or 1‐M phosphoric acid as required.

### 2.4. Construction of the Calibration Curve

Five working standard solutions in the range of 5–15 μg/mL were prepared by appropriate dilution from the standard stock solution with respective dissolution medium (pH 1.2, pH 4.5, and pH 6.8 and 50% v/v methanolic NaOH) as solvents [31, 32]. As described in the validated adopted method, the solutions were filtered using a 0.2‐μm membrane filter before being injected into the HPLC system. The resultant plot of the peak area versus concentration was used for later analysis by deriving the corresponding concentrations by referring to the calibration curve having the regression equation *Y* = 11934*X* − 3317 with a limit of detection and quantitation of 0.953 μg/mL and 3.170 μg/mL, respectively.

### 2.5. Specificity of the Dissolution Method

The specificity of the dissolution method was tested by examining for any peak interference from the dissolution medium in comparison with standard levothyroxine and in comparison with the four tested formulations. Aliquots of the three‐dissolution media were injected in the HPLC system. Chromatograms revealed the absence of any interference from the dissolution media where no extra peaks were observed.

Aliquots of the standard levothyroxine prepared by dilution using dissolution media were also injected in the HPLC system. Also, for each pharmaceutical formulation, five tablets were weighed and finely powdered. Around half the weight (equivalent to 250 μg of levothyroxine) was transferred to a 25‐mL volumetric flask, dissolved in the dissolution media. The solutions were then injected into the LC system and chromatographed. The peak area values were measured, and the corresponding concentrations in the tablets were derived by referring to the calibration graph to ensure that each tablet contained its labeled amount. There was no significant effect on the standard levothyroxine sodium peaks in terms of measurement, tailing and retention time. Also, high % recovery was established for the tested formulations in the dissolution media (Table 2).

**Table 2:** Recovery analysis for levothyroxine sodium tablets in the dissolution media.

Formulation	Mean % recovery ± SD		
	1.2	4.5	6.8
Euthyrox100® (old formulation)	99.382 ± 2.314	98.615 ± 3.752	97.591 ± 4.194
Euthyrox100® (new formulation)	100.063 ± 3.806	99.472 ± 2.941	99.626 ± 2.923
Eltroxin®	98.529 ± 2.843	97.028 ± 3.275	96.438 ± 2.126
Levothyroxine Sandoz®	99.391 ± 2.974	97.794 ± 3.058	98.712 ± 3.758

### 2.6. Dissolution Studies

Dissolution was carried out using a Copley dissolution tester. The dissolution bath was set to 37 ± 0.5°C, and 500 mL of the dissolution media was added to each vessel; the rotor speed was set to 50 RPM and allowed to equilibrate for at least 1 hour. The dissolution testing was carried out according to the USP paddle method in three different dissolution media: buffer pH 1.2, buffer pH 4.5 and buffer pH 6.8.

All the dissolution studies were performed in six replicates (vessels) for each pharmaceutical formulation, and tablets were released into the relevant vessels simultaneously. Samples (1 mL) were taken from each vessel at 5, 10, 20, 30, 45, and 60 min and replaced with equal volumes of fresh buffer equilibrated to 37°C after each sample withdrawal. Samples were filtered through 0.2‐μm disposable filters. The withdrawn samples at each time point during the dissolution studies were diluted in a ratio of 1:1 with 10 μg/mL of standard levothyroxine as a way of employing the standard addition method. Levothyroxine concentrations were analyzed using a validated and published anion‐exchange HPLC method using HiQ Sil NH_2_ column and a mobile phase composed of HPLC‐grade acetonitrile:acetate buffer in the ratio of (55:45, %v/v) at a flow rate of 1.2 mL/min and at a wavelength of 252 nm [31]. As such, a volume of 20 μL of the diluted samples was then directly injected in the LC system and the peak area of the resultant signal from the samples was measured and the concentration was computed from the calibration curve.

### 2.7. Data Analysis

The dissolution profiles were constructed by plotting the % levothyroxine released versus time. The dissolution data were compared using model‐dependent and model‐independent methods. Various model‐dependent methods (zero order, first order, Higuchi, Korsmeyer–Peppas, Hixson–Crowell, Weibull, and Baker–Lonsdale) were applied to determine the best model that describes levothyroxine release kinetics from the tested formulations [33–35]. The dissolution profiles of the tested formulations were evaluated by fitting the experimental data to the models (Table 3) [36, 37]. The model‐independent method was used to compare the dissolution profiles for the different formulations. This method is based on the calculation of the fit factors (difference factor (*f*_1_) and the similarity factor (*f*_2_) which are recommended for dissolution profile comparison as described in the FDA’s guidelines for industry. The difference factor (*f*_1_) calculates the percentage difference (dissimilarity) between two dissolution profiles at each time point and is a measure of the relative error between the two curves, as described in equation (1), whereas the similarity factor (*f*_2_) is the logarithmic reciprocal square root transformation of the sum of squared error and is a measurement of the similarity in the percent dissolution between the two curves, as described in equation (2) [35, 38–40]. According to the FDA’s guidelines, *f*_1_ values between 0 and 15 and *f*_2_ values between 50 and 100 show the similarity of the dissolution profiles and are suggestive of an average difference of no more than 10% at each sample time point [40].

(1) f1=∑t=1nRt−Tt∑t=1nRt×100,

(2) f2=50 log1+1n∑t=1nRt−Tt2−0.5×100.

**Table 3:** Mathematical models used to evaluate the release kinetics of the drug [36, 37].

Zero order	*Q*_*t*_ = *Q*_0_ + *k*_0_ *t*
First order	ln *C*_*t*_ = ln *C*_0_ − *k*_1_ *t*
Higuchi	$Q_{t}=k_{H}\sqrt{t}$
Korsmeyer–Peppas	*Q*_*t*_/*Q*_*∞*_ = *K*_*k*_ *t* ^*n*^
Hixon–Crowell	$C_{0}^{13/}-C_{t}^{13/}=K_{s}t$
Weibull	log[−ln(1 − *Q*_*t*_/*Q*_*∞*_)] = *β* log(*t* − *T*_*d*_) − log *α*
Baker–Lonsdale	$\left(32/\right)\left\{1-{\left(1-\left[Q_{t}/Q_{\infty }\right]\right)}^{23/}\right\}-\left(Q_{t}/Q_{\infty }\right)=K_{b}t$

*Note:* *Q*_*t*_: amount of drug released in time *t*, *Q*_0_: initial amount of drug in the dissolution medium, *C*_*t*_: amount of drug remaining at time *t*, *C*_0_: initial amount of drug in the tablet, *Q*_*t*_/*Q*_*∞*_: fraction of drug released at time *t*, *k*_0_, *k*_1_, *k*_*H*_, *K*_*k*_, *K*_*s*_, and *K*_*b*_: release rate constants, *n*: the release exponent, *β*: shape parameter, *α*: scale parameter *α* = ${\left(T_{d}\right)}^{\beta }$, and *T*_*d*_: time parameter.

In the above equations, *n* is the number of dissolution sample time points, and *R*_*t*_ and *T*_*t*_ are the percent dissolved at each time point, *t*, for the reference product and test dissolution profiles, respectively.

For the present study, Euthyrox new formulation was used as the reference product, and the three other formulations were compared with it using *f*_1_ and *f*_2_. As well, the effect of pH on dissolution was studied by plotting the % levothyroxine dissolved at 30 and 60 against pH.

## 3. Results and Discussion

The lack of definitive data on the BCS levothyroxine classification and formulation variability effect on the bioavailability of levothyroxine has led to developing a biowaiver study to assess these factors in different formulations. The dissolution of a drug from its formulation is an essential predictor for its absorption and bioavailability. The drug must be first solubilized in the GI fluids to be absorbed. Accordingly, the dissolution profile can provide information about its bioavailability. So, when different drug formulations exhibit similar dissolution profiles, this would suggest that they may have similar in vivo performance in terms of absorption and bioavailability.

Dissolution testing for levothyroxine, as described in the USP47, can be used as a one‐point quality control standard. An amount of levothyroxine released not less than 70% of the labeled amount in 45 min in acidic medium is considered fitting to the acceptance criteria [41].

**Figure 1:**
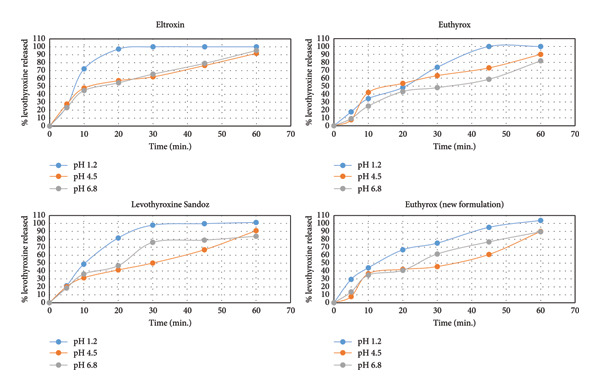
Dissolution profiles of Eltroxin, Euthyrox, levothyroxine Sandoz, and Euthyrox new formulation in three different dissolution media (pH 1.2, 4.5, and 6.8).

The dissolution profiles of Euthyrox100 (old and new formulations), Eltroxin 100, and Sandoz generic levothyroxine sodium in the three‐dissolution media are shown in Figure 1. These dissolution profiles show that the mentioned criteria, more than 70% is released in 45 min, was satisfied by the four tested formulations, signifying their similarity as a primary evaluation.

Keeping in mind, individual differences and physiological variability in the pH of the GIT between patients can also influence levothyroxine absorption. So, dissolution studies for the three formulations in comparison to Euthyrox new formulation as reference were assessed. The difference factor (*f*_1_) and the similarity factor (*f*_2_) were calculated from the means of the % levothyroxine dissolved at each time point using equations (1) and (2) mentioned earlier and the results are given in Table 4. According to the f‐criteria, it can be concluded that the Euthyrox new formulation dissolution profile is only similar to the old one at pH 1.2, while all other dissolution profiles were not similar. These differences in the dissolution profiles could be attributed to existing differences in shape and size of each pharmaceutical formulation as well as the amount and type of excipients used.

**Table 4:** Difference factor (*f*_1_) and similarity factor (*f*_2_) for Eltroxin, Euthyrox, and levothyroxine Sandoz using Euthyrox new formulation as the reference product in three different dissolution media (pH 1.2, 4.5, and 6.8).

Dissolution medium pH	1.2		4.5		6.8	
Factor	*f*_1_	*f*_2_	*f*_1_	*f*_2_	*f*_1_	*f*_2_
Eltroxin	20.19	65.93	28.23	56.48	13.58	44.50
Euthyrox	**14.99**	**50.25**	16.47	48.68	18.25	50.57
Levothyroxine Sandoz	16.19	55.51	10.15	40.18	11.42	42.31

*Note:* The bold values here are considered accepted values for *f*_1_ and *f*_2_. *f*_1_ should be less than 15 and *f*_2_ greater than 50, and this applies together only for the bolded values.

**Figure 3:**
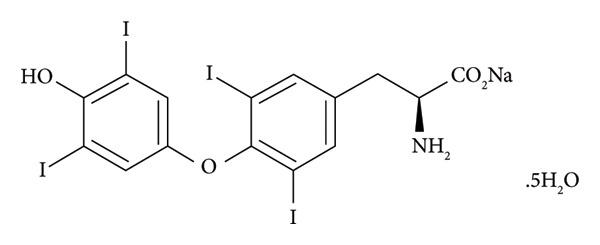
Chemical structure of levothyroxine.

The presented dissolution profiles in Figure 1 show that pH has a significant role on levothyroxine’s dissolution as the % levothyroxine released changed with the change of the dissolution medium. This effect was demonstrated in Figure 2 by plotting the % levothyroxine released at 30 and 60 mins against pH. For all drug formulations, the fastest and almost complete dissolution was obtained in the dissolution medium having the pH of 1.2. After 60 min, at pH 4.5 and 6.8, incomplete dissolution was observed, where differences between the obtained dissolution profiles were notable at the early time points. Slower release was seen at pH 4.5. This can be supported by that levothyroxine is an amphoteric molecule containing three ionizable functional groups, two acidic (carboxylate and phenolate) and one basic amine (Figure 3). As such, the pH value controls the ionization and has a great impact on levothyroxine’s solubility and accordingly on its dissolution from the tablets [42].

Figure 2Comparison of % levothyroxine released from Eltroxin, Euthyrox, levothyroxine Sandoz, and Euthyrox new formulation at dissolution media pH 1.2, 4.5, and 6.8 at the end of (a) 30 min and (b) 60 min.

**Figure:**
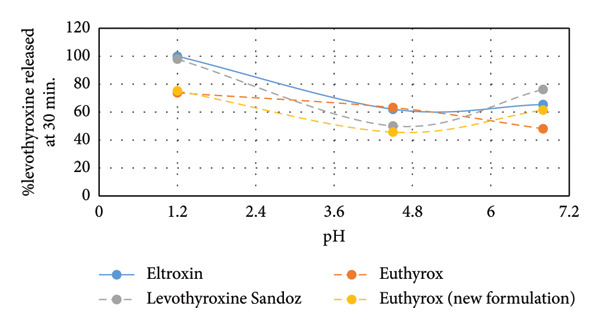
(a)

**Figure:**
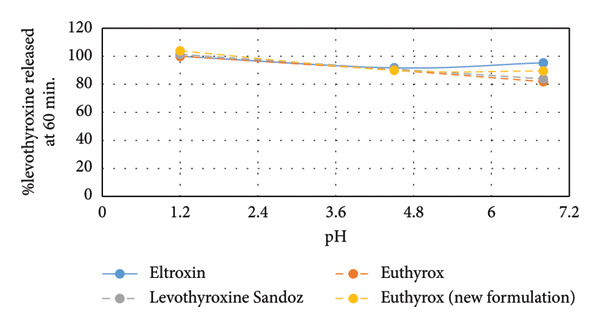
(b)

Theoretically, levothyroxine is highly soluble at pH values below 2.2 and above 10.1. So physiologically, a levothyroxine tablet is expected to have the highest dissolution in the stomach where the pH ranges from 1 to 2. However, dissolution is influenced by other physical factors including the particle size, particle distribution, excipients used, and manufacturing process [43]. Thus, the bioavailability is highly dependent on the extent to which these factors affect the dissolution of levothyroxine, especially that the excipients in levothyroxine tablets constitute the bulk of the tablet; levothyroxine constitutes less than 0.1% of the tablet weight. Dissolution profiles in different pH media were used to compare and evaluate the release, bioavailability, and similarity between the four levothyroxine brands.

Complete dissolution was seen at pH 1.2 whereas slower release and incomplete dissolution were seen at pH 4.5 and 6.8. These results signify that levothyroxine’s dissolution is pH sensitive. Thus, it can be concluded that in vivo difference and what patients report on a difference in the effect arise at higher pH values. This can be attributed to the molecule itself as it has the property of self‐aggregation and lowering the dissolution by having a low intrinsic solubility at higher pH values. Also, it can be due to individual differences and patient noncompliance and adherence to the mode of administration of this drug (on empty stomach and not to eat until 30–60 min after the tablet intake). Hence, food or medication intake that may decrease the acidity will result in decreased dissolution and less drug absorption, requiring higher dosing of levothyroxine [40].

The dissolution profiles of the four levothyroxine formulations were also used to assess the drug release model and kinetics. The dissolution data at the three pH values (1.2, 4.5, and 6.8) were fitted to zero‐order, first‐order, Higuchi, Korsmeyer–Peppas, Hixson–Crowell, Weibull, and Baker–Lonsdale models (Table 5). The correlation coefficient (*R* ^2^) was the parameter used to choose the most suitable model. The higher *R* ^2^ represents a model that is more applicable to the release curve.

**Table 5:** Comparative release kinetics model for the four tested levothyroxine tablets at pH 1.2, 4.5, and 6.8.

Dissolution media and product	Correlation coefficient (*R* ^2^)						
	Zero order	First order	Higuchi	Korsmeyer–Peppas	Hixon–Crowell	Weibull	Baker–Lonsdale
pH 1.2							
Eltroxin	0.9305	0.9622	0.9221	0.9055	0.9844	**0.9889**	0.9072
Euthyrox	**0.985**	0.9658	0.9525	0.9816	0.9192	0.9736	0.9772
Euthyrox (new formulation)	0.9055	0.9537	**0.9963**	0.9932	0.9827	0.9668	0.9486
Levothyroxine Sandoz	0.9591	0.9722	0.948	0.9232	0.9765	**0.9966**	0.7961
pH 4.5							
Eltroxin	0.8565	0.9442	**0.9787**	0.955	0.9512	0.9389	0.7803
Euthyrox	0.8743	**0.9561**	0.9481	0.8161	0.9567	0.8921	0.8095
Euthyrox (new formulation)	0.9177	0.8567	**0.9197**	0.8441	0.9058	0.8737	0.8747
Levothyroxine Sandoz	0.9611	0.8937	0.9684	0.9793	0.9456	0.914	**0.9881**
pH 6.8							
Eltroxin	0.8989	0.9285	**0.9897**	0.9617	0.9684	0.9473	0.8313
Euthyrox	0.9489	0.9428	0.9591	0.9457	0.9624	**0.9634**	0.9253
Euthyrox (new formulation)	0.9454	0.9804	0.9754	0.9507	**0.9901**	0.9681	0.9342
Levothyroxine Sandoz	0.8589	0.9351	0.9574	0.9496	0.9159	**0.9638**	0.8392

*Note:* The bold values represent the highest correlation coefficient for each formulation in the different dissolution media when fitted to the different kinetic models.

At the three used pH values (1.2, 4.5, and 6.8), there was no single release kinetic model that describes the release of levothyroxine sodium from all the formulations. Based on the difference in the excipient composition of each formulation, particle size, crystal form, and manufacturing processes, each brand showed a different release model at the different pH values.

At pH 1.2, the highest regression coefficient (*R* ^2^) for Eltroxin (0.9889) and levothyroxine Sandoz (0.9966) was observed for the Weibull model. The regression equations were *Y* = 1.8083*X* − 1.7677 and *Y* = 1.499*X* − 1.6746, which show that the drug release from the tablet starts slows followed by an accelerated release due to structural changes in the tablet like swelling or erosion, whereas Euthyrox old formulations showed zero‐order release kinetics with *R* ^2^ values 0.985. The zero‐order system describes a constant drug‐release rate from the tablet per unit time regardless of the provided or remaining dose in the tablet. While the new modified Euthyrox formulation follows the Higuchi model. This implies the presence of an excipient effect forming an insoluble matrix in which levothyroxine diffuses out of the matrix. According to the Higuchi model, the rate at which levothyroxine is released is proportional to the square root of time. This shows that as time proceeds, the rate of release decreases, leading to gradual release into the dissolution medium [34, 36, 44].

In the acetate buffer (pH 4.5), Eltroxin and Euthyrox new formulation showed a Higuchi release model, whereas Euthyrox old formulation followed first‐order kinetics. The first‐order release rate is concentration dependent, where levothyroxine is released from the remaining amount of drug in a porous matrix formulation, while the Baker–Lonsdale model describes the release from levothyroxine Sandoz. The Baker–Lonsdale model describes drug release from a spherical matrix where the inert matrix does not swell nor erode, and only the drug is released via diffusion [34, 36, 44].

At pH 6.8, Eltroxin followed the Higuchi model. Euthyrox (old formulation) and levothyroxine Sandoz showed a Weibull kinetic release, whereas the highest *R* ^2^ value for the Euthyrox new formulation was observed when fitted to the Hixon–Crowell equation model. Levothyroxine sodium is released from the system where there is a change in surface area and particle size during the dissolution process [34, 36, 44].

## 4. Conclusion

In levothyroxine pharmaceutical tablets, the excipients constitute the bulk (≥ 99.9%). Accordingly, these excipients have a major effect on the stability and dissolution of the tablets and should be carefully considered during manufacturing. Although the excipients are often considered inert and pharmacologically and physiologically inactive, however there was a significant effect due to levothyroxine small concentrations in the tablets. Their effect was demonstrated in four different levothyroxine formulations’ dissolution profiles. The use of a validated anion‐exchange HPLC method allowed the quantitation of levothyroxine dissolved in the three tested dissolution media for the four different tablet formulations. Dissolution data were used to assess a kinetic release model for levothyroxine sodium from its tablets; however, the release varied from one formulation to another at different pH values. The four levothyroxine formulations showed dissimilar dissolution profiles, indicating that these results are formulation dependent. Such difference in dissolution affects the absorption and bioavailability of levothyroxine, which justifies the different physiological response in patients when switching between different formulations. Also, it was found that the dissolution of levothyroxine is pH sensitive. These data support the idea of aggregation and low intrinsic dissolution of the levothyroxine molecule, causing to have molecules with a greater particle size that cannot pass through intestinal membrane or fit the protein transporter or even dissolve at later sites in the intestine (where the drug is not absorbed), limiting its absorption and leading to the erratic bioavailability signifying low permeability. In brief, levothyroxine technically belongs to Class III, but practically, in vivo, due to its complex behavior in dissolution, it is preferred not to classify it as Class III. In conclusion, the dissolution of levothyroxine is critical for its bioavailability and may be the contributing factor for the bioequivalence problem between various formulations. Therefore, it should be analyzed and regulated beyond the BCS classifications as a NTI drug strictly requiring bioequivalence testing for generic approval and substitution.

## Conflicts of Interest

The authors declare no conflicts of interest.

## Funding

No funding was received for this study.

## Data Availability

The data that support the findings of this study are available from the corresponding author upon reasonable request.
